# Antimicrobial, antibiofilm and antioxidant activities of bioactive secondary metabolites of marine *Scarus ghobban* gut-associated *Aspergillus niger*: In-vitro and in-silico studies

**DOI:** 10.1038/s41598-025-06605-6

**Published:** 2025-07-02

**Authors:** Hagar Abdellatief, Amira E. Sehim, Amany M. Emam, Mahmoud Amer, Sawsan Dacrory, Amr H. Hashem

**Affiliations:** 1 Botany and Microbiology Department, Faculty of Science, Benha University, Benha, 13518 Egypt; 2 Cellulose and Paper Department, National Research Centre, Giza, 12622 Egypt; 3 Botany and Microbiology Department, Faculty of Science, Al-Azhar University, Cairo, 11884 Egypt

**Keywords:** Fish gut associated -fungi, Antibacterial, Antibiofilm, Gas cromatography–mass spectrometry, Transmission electron microscopy, Docking study, Biotechnology, Microbiology

## Abstract

Fungal extracts have garnered significant interest in recent years for their diverse applications in pharmaceutical field. This research focused on isolating fungi from the gut of *Scarus ghobban* for the first time and evaluate their biological activities *Aspergillus niger* was successfully isolated and identified using morphological and molecular techniques. Gas chromatography–mass spectrometry (GC-MS) analysis of the ethyl acetate extract (EA) of *A. niger* revealed eight compounds, with diisooctyl phthalate (54.32%) and 1,2-benzenedicarboxylic acid, bis (2-methoxyethyl) ester (26.32%) as the most abundant. High-performance liquid chromatography (HPLC) analysis identified catechol (15.41 µg/mL) and syringenic acid (13.25 µg/mL) as prominent phenolic compounds in the extract. The EA extract exhibited significant antibacterial activity toward pathogenic bacterial strains, with the highest inhibition zone (32 ± 0.1 mm) and minimum inhibitory concentration (MIC) of 7.8 µg/mL against *Bacillus subtilis*. Additionally, it showed antifungal activity against *Candida tropicalis* (MIC 7.8 µg/mL) and *Candida albicans* (MIC 31.25 µg/mL). The extract also demonstrated potential antibiofilm activity against *Salmonella typhimurium*, *Staphylococcus aureus*, *Enterococcus faecalis*, and *Escherichia coli*, with inhibition percentages exceeding 87%. Moreover, it exhibited potent antioxidant activity IC50 8.17 µg/mL. Transmission electron microscopy revealed severe structural damage in *B. subtilis*, emphasizing the extract’s antibacterial effectiveness and potential for therapeutic applications. Eventually, docking studies and computational calculations have been utilized to demonstrate the reactivity of the molecules. In conclusion, the ethyl acetate extract of *A. niger* from gut of *S. ghobban* demonstrates significant antibacterial, antibiofilm, and antioxidant activities, highlighting its potential as a valuable resource for developing effective antimicrobial agents and therapeutic applications.

## Introduction

Pathogenic bacterial contamination has emerged as a major public health concern in the food, medical, agricultural, and environmental sectors, resulting in severe human diseases and financial losses on a worldwide scale^1–3^. It’s believed that 80% of pathogenic infections are attributed to difficult-to-eliminate biofilms, which significantly enhance the resistance of pathogenic and spoilage bacteria to conventional antibiotics and disinfectants, rendering them 10 to 1000 times more resilient against these treatments. This underscores the critical need for novel therapeutic approaches, such as those derived from natural sources like fungal extracts, to effectively combat biofilm-associated infections^4^. It’s important and challenging to treat persistent medical infections, rapid food spoilage and associated diseases attributable to repeated contamination of bacterial biofilm^5^. For removal or destruction of biofilm, chemical techniques have been widely studied, but there are many important limitations including high toxicity, low efficacy and drug resistant, especially antibiotic resistance^6^. This emphasizes how vital it is to create antibiofilm agents that are safe for the environment. In addition to their ecological and economic significance, coral reef habitats are well-known for their high biodiversity^7^. Coral reef fish, one of the most significant communities in coral reef ecosystems, are vital to the reef resilience and functioning of the ecosystem^8^. The intestinal tract of fish contains an abundance of microorganisms where a complex symbiotic relationship between host and microorganisms was formed in a long-term natural evolution process^9,10^. A reproductive environment for intestinal microorganisms is provided by the fish gut. Furthermore, immune metabolism, nutrition, growth and physiological health of the host depend on these intestinal microbes^11,12^.Therefore, Our knowledge about the complex interaction between the host and the microorganisms that inhabit the fish gut greatly enriches due to study the microflora inhabiting the fish gut in many species of coral reef fish^13^.

In marine environments, *Aspergillus* is a genus of fungi that is widely found^14,15^. *Aspergillus fumigatus*,* A. niger*,* A. versicolor*,* A. flavus*,* A. ochraceus*,* A. ticus*,* and A. terreus*, etc. are examples of prevalent species. Azolones, flavonoids, steroids and other bioactive natural compounds can be produced by marine *Aspergillus* which is a valuable source^16–18^. These metabolites exhibit a variety of biological activities including lipid-lowering, antibacterial anticancer, antiviral, anti-inflammatory, and anti-diabetic effects. They also have a variety of different structures^19–22^. According to recent studies, *A. niger* has been confirmed to be an essential source of bioactive natural compounds with a variety of biological activities^23^. Rasouli et al. reported that *A.niger* has antibiofilm activity on clinical *staphylococcus epidermidis* and *Pseudomonas aeruginosa*^24^. The aim of this study was to isolate fungi from the gut of *Scarus ghobban* fish and evaluate their antibacterial, antibiofilm, and antioxidant activities, with the goal of exploring their potential applications in pharmaceutical applications.

## Materials and methods

### Collection of fish sample

*Scarus ghobban* fish was collected from the Suez Gulf region, Egypt, situated along the Red Sea, (28° 45′ 0″ N latitude and 33° 0′ 0″ E) in March 2023. The Gulf of Suez extends about 314 km with an average depth of 40 km. The collected fish was directly placed in sterile Ziploc plastic bags, an ice box was used for transportation to the laboratory, and maintained at 4 ^o^C for fungal isolation.

### Isolation of fungi from *Scarus ghobban* gut

After the fish was collected, it was transferred in a dissecting tray and its entire body was cleaned with 75% alcohol. After using dissecting scissors to cut the fish in an upward arc along the anus, The gut was removed aseptically and put into a 15 mL centrifuge tube (Eppendorf, Germany) and diluted with 3 ml of sterile water. An analog vortex mixer (OHAUS Corporation, United States of America) (OHAUS, USA) was used to shake the tubes vigorously. On sterile petri dishes with Glucose Yeast Peptone Agar (GYPA) medium (0.5% glucose, 0.1% yeast extract, 0.5% peptone, and 2% agar) containing chloramphenicol (0.2 g/l) to inhibit bacterial growth, a 100 µL dilution of the sample was applied. A sterilized plates culture media was used as a negative control and plated under the same conditions to ensure that no contamination occurred during the isolation process. At 25 ^o^C, plates were incubated and the growth of fungal hyphae was observed every day. In order to get pure cultures, fungal colonies found on the medium were recultured into fresh Potato Dextrose Agar (PDA) (20% potato, 2% glucose, 2% agar). The isolated fungal strain was kept for further investigation at 4 °C^25^.

### Morphological and molecular identification of the fungal isolate

The Morphological identification of the fungal isolate was performed by detecting the macroscopic features including color, texture, and appearance as well as microscopic features by utilizing a light microscope. Additionally, the examined fungus was molecularly identified using 28 S rRNA gene sequencing in accordance with the manufacturer’s methodology used by Sigma Scientific Services Company (Giza, Egypt). National Center for Biotechnology Information’s nucleotide Basic Local Alignment Search Tool (NCBI n-BLAST) search software was utilized to compare the sequence with similar sequences that were obtained from DNA databases https://www.ncbi.nlm.nih.gov/genbank/. In MEGA version 11, phylogenetic and evolutionary studies were carried out^26,27^.

### Extraction of fungal active compounds

In 2000-mL Erlenmeyer flasks, the fungal isolate was placed in 1000 mL of PDB and maintained at 28 ^o^C for 21 days under static conditions. To get the fungal mycelia, the broth medium was filtered. In order to extract the filtrate with ethyl acetate, it was combined with an equivalent volume of the solvent, shaken on a vortex shaker for 10 min, and then allowed to settle for 5 min to create two separate layers. Then, the ethyl acetate layer was separated using a separating funnel and evaporated in an oven set at 60 °C. After dissolving the resultant crude fungal extract in 1% Dimethyl sulfoxide (DMSO) to reach the last concentration of 1 mg/mL, it was kept for use in subsequent studies at -20 °C^28^.

### Gas chromatography-mass spectrometry (GC-MS)

The chemical composition of the EA extract of *A. niger* was analyzed using a trace Gas Chromatography-Triple Quadrupole  (GC-TSQ) mass spectrometer (Thermo Scientific, Texas, USA). The setup includes a direct capillary column TG-5MS. The ion source temperature was maintained at 200 °C, and the mass spectra of the extract were compared with those in the WILEY 09 and National Institute of Standards and Technology (NIST) 14 mass spectrometry databases^29,30^.

### High-performance liquid chromatography (HPLC) analysis

By Using Agilent series 1100 HPLC technique (Agilent, USA), the analysis of phenolic compounds of EA fungal extract was carried out. HPLC system included solvent degasser, auto-sampling injector, two 1100 series Liquid Chromatography) LC (pumps, ChemStation software, as well as UV/Vis detector adjusted at 250 nm. The gradient program began with 100% Solvent B (1:25 solution of acetic acid in water) for 3 min, then 50% Solvent A (methanol) for 5 min, then elevated to 80% Solvent A for two minutes, and finally decreased to 50% for the final 5 min. With injection volumes of 25 µL, the separation was performed at 25 °C and the flow rate of the solvent was 1 mL/min^31,32^.

### Antibacterial assay

In accordance with Clinical and Laboratory Standards Institute (CLSI) guideline M51-A2^33^, the agar well diffusion method was used to assess the antibacterial activity of the EA extract of *A. niger* against *Escherichia coli* ATCC 8793, *Salmonella typhi* ATCC 6538, *Klebsiella pneumoniae* ATCC13883, *Staphylococcus aureus* ATCC 25923, *Bacillus subtilis* ATCC 6633 and *Enterococcus faecalis* ATCC 29212. Tested bacterial strains were cultured individually on Muller Hinton agar plates. 100 µL of EA fungal extract (1 mg/mL), gentamicin (1 mg/mL) and DMSO was put in each well (7 mm). The plates were refrigerated for 2 h and then incubated for 24 h at 37 °C. After that, the diameter of the inhibition zone was measured^34,35^.

### Antifungal assay

The antifungal activity of EA extract of *A. niger* against unicellular fungi including (*Candida tropicalis* and *Candida albicans*) using agar well diffusion method. fungal suspensions were regularly dispersed across Sabouraud Dextrose Agar (SDA) plates individually. After that, 100 µL of EA fungal extract (1 mg/mL), positive control fluconazole (1 mg/ mL) and DMSO as negative control were added to agar wells (7 mm) separately. For 3 days at 30 °C, All plates were incubated then we measured the diameter of inhibition zones^34^.

### Determination of minimum inhibitory concentration (MIC), minimum bactericidal concentration (MBC), and minimum fungicidal concentration (MFC)

The broth microdilution method (CLSI, 2020) was used to determine the MIC and MBC of EA extract of *A. niger*. The MIC index (MICi) was calculated using Eq. 1, which provided clarification on the interpretation of the effect of tested fungal extract, whether bacteriostatic/ fungistatic or bactericidal/ fungicidal. The extract exhibited bacteriostatic/ fungistatic effect when its MICi value was ≥ 4, and bactericidal/ fungicidal activity when it was ≤ 2^36,37^.

1 $$\bf \:\:{\text{M}\text{I}\text{C}}_{\text{i}}=\frac{\text{M}\text{B}\text{C}/MFC}{\text{M}\text{I}\text{C}}$$

### Antibiofilm assay

Antibiofilm activity of EA extract of *A. niger* was evaluated using 96-well polystyrene flat-bottom plates. In summary, each microplate well received 300 µL of newly inoculated trypticase soy yeast (TSY) broth at 10^6^ CFU/mL. Sublethal extract concentrations of 75, 50, and 25% of the MIC were then added to the microplates. Control wells contained just medium and no extract^38,39^. The inhibition of bacterial film (IBF) formation was calculated using the following equation:

$$\:\:\:\%\text{I}\text{B}\text{F}=1-\frac{\text{T}\text{r}\text{e}\text{a}\text{t}\text{e}\text{d}\:\text{A}\text{b}-\text{B}\text{l}\text{a}\text{n}\text{k}\:\text{A}\text{b}}{\text{C}\text{o}\text{n}\text{t}\text{r}\text{o}\text{l}\:\text{A}\text{b}-\text{B}\text{l}\text{a}\text{n}\text{k}\:\text{A}\text{b}}\times\:100$$

Blank Ab represented the absorbance of the media only. While the control Ab showed bacteria absorption without any treatment with fungal extract. Also, treated Ab represented the absorbance of the test organism after treatment.

### Transmission electron microscopy

To examine the impact of EA extract from *A. niger* on the ultrastructure of the most susceptible bacteria, bacterial cells were centrifuged at 4000 rpm for 10 min. Following a wash with distilled water, the samples underwent post-fixation in a potassium permanganate solution for 5 min after being fixed in 3% glutaraldehyde and rinsed in phosphate buffer at room temperature. Following 15 min of dehydration in each ethanol dilution, ranging from 10 to 90%, the samples underwent an additional 30 min of dehydration in absolute ethanol. Samples were infiltrated with acetone and epoxy resin in a graded sequence, followed by infiltration with pure resin, and ultrathin sections were obtained using copper grids. Subsequently, uranyl acetate was employed following lead citrate to achieve twofold staining of the sections. Ultimately, stained sections were analyzed at RCMB, Al-Azhar University, utilizing a JEOL-JEEM 1010^40,41^.

### Antioxidant activity using DPPH

The evaluation of antioxidant activity of EA extract from *A. niger* was conducted using the DPPH assay, following the methodology outlined in references^42,43^. One milliliter of a 0.1 mM DPPH solution in ethanol was mixed with three milliliters of fungal extract at concentrations varying from 1,000 to 1.95 µg/mL. The solution was permitted to equilibrate at ambient temperature for 30 min following thorough agitation. Following that, we measured the absorbance at 517 nm with a Milton Roy UV-VIS spectrophotometer. Ascorbic acid was utilized as the reference standard. The percentage of inhibition or DPPH scavenging activity (%) is determined using the formula below:

$$\text{D}\text{P}\text{P}\text{H}\:\text{s}\text{c}\text{a}\text{v}\text{e}\text{n}\text{g}\text{i}\text{n}\text{g}\:\text{e}\text{f}\text{f}\text{e}\text{c}\text{t}\:\left(\text{\%}\right)=\:\frac{\text{A}0-\text{A}1}{\text{A}0}\times\:100$$

where A0 and A1 stand for the control (DPPH solution) and sample absorbances, respectively.

### Ferric reducing antioxidant power (FRAP) assay

To evaluate the impact of solvent polarity on the total reducing capacity of the extracts, modified potassium ferricyanide and trichloroacetic acid approach^44^ was used and adaptable for microplate application^45^. Ferric reducing antioxidant power (FRAP) will henceforth be called total reducing power (TRP). 40 mL of the sample was put into each labeled Eppendorf tube, which was then diluted with 50 mL of 0.2 mol/L sodium phosphate dihydrate (Na2HPO4·2H2O) buffer and 50 mL of 1% potassium ferricyanide (K3Fe (CN)6), as well as 50 mL of 10% trichloroacetic acid. For 10 min, the mixture was centrifuged at 3,000 rpm. Subsequent to centrifugation, 33.3 mL of 1% ferric chloride (FeCl3) was mixed with 166.66 mL of each sample’s supernatant that had been transferred to a 96-well plate. The absorbance was measured at 630 nm by using microplate reader (Biotek ELX800; Biotek, Winooski, VT, USA). The positive control was ascorbic acid (1 mg/mL), while the negative control was DMSO. The results were presented as microgram of ascorbic acid equivalent (AAE) per milligram of extract.

### Computational procedures

The Gaussian 09 W program was employed to conduct calculations of Density Functional Theory (DFT) using the hybrid functional B3LYP (Becke’s three-parameter hybrid functional combined with the BLYP correlation functional) and the 6-31G(d) basis set, employing the Berny method^46^.

### Molecular modeling and docking

The molecular modeling of compounds 1, 2-benzen dicaboxylic acid, bis (2-methoxyethyl) ester (comp. 2) and 5-hydroxy-2,2-dimethyl-5,6-bis-(2-oxopropyl)-cyclohexanone (comp. 5) against *B*. *subtilis* (ATCC 6633), *E. faecalis* (ATCC 29212) and *S.aureus* (ATCC 6538). As well, anticancer activity breast cancer MCF-7 and hepatic HepG2 has studied and fabricated using standard bond lengths and energy, with the Auto Dock Vina and detected by Discovery Studio Client (version 4.2)^47^.

## Results and discussion

### Isolation and identification of fish gut fungus

**Fig. 1:**
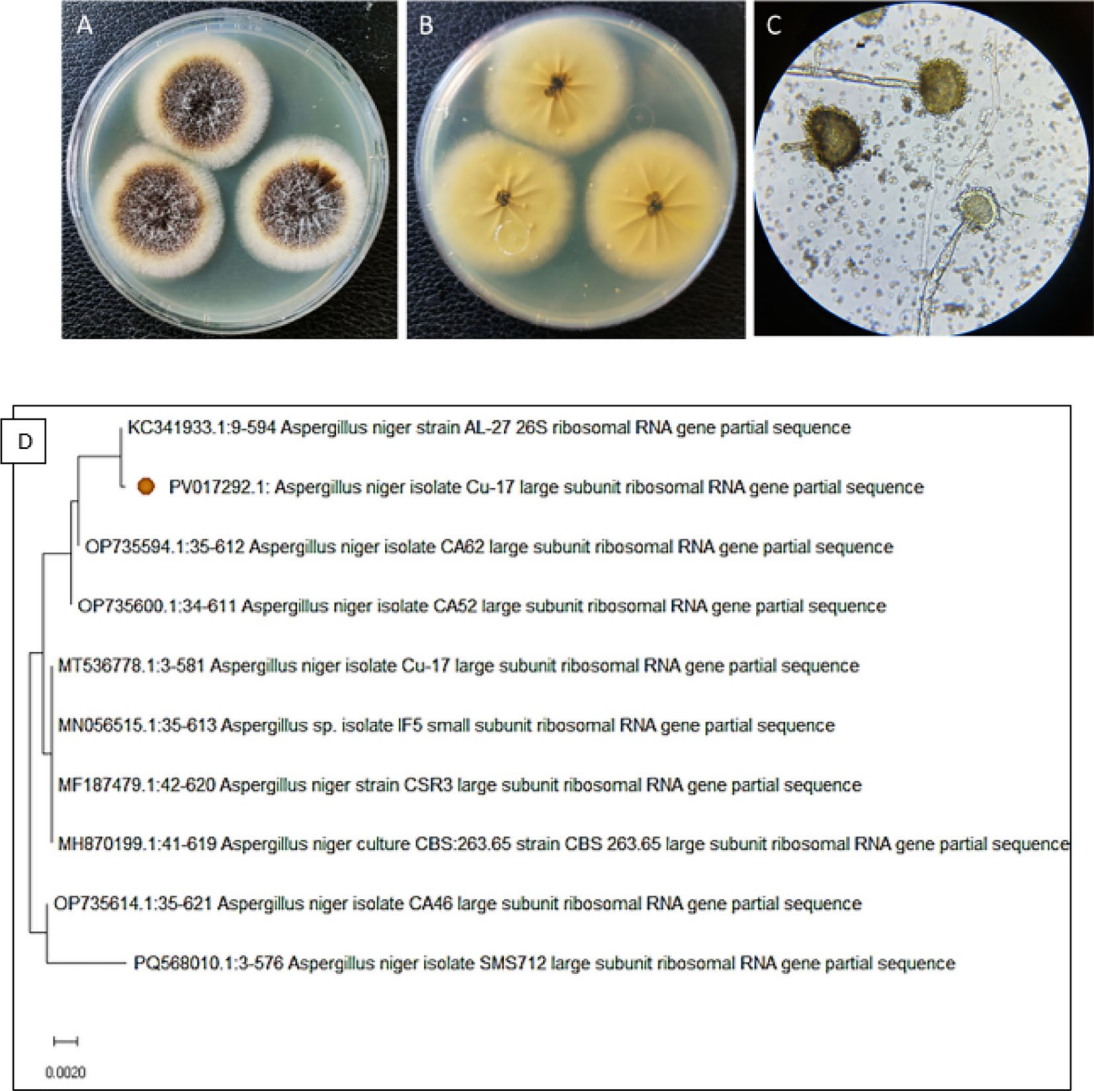
(**A**) surface colony of *A. niger* on PDA grown; (**B**) reverse colony; (**C**) light microscope showing conidiophore, and conidia of *A. niger* (400X); (**D**) Phylogenetic tree of *A. niger*.

In this study, fungal isolate S1 was isolated from the gut of *S. ghobban.* Identification of this fungal isolate was performed using morphological and molecular techniques. The morphological examination indicated that the colonies proliferate swiftly, attaining a size of 40 mm within 4 days at 28 ^o^C on PDA, characterized by powdery black colonies with a faint yellow reverse, as illustrated in (Fig. 1). Microscopic examination revealed that mycelium is hyaline and septate, whereas, conidiophores are non-septate, erect, and smooth-walled, globose vesicles containing hyaline conidia. Conidia are smooth and spherical. Then, molecular identification was carried out using 28 S rRNA gene. Molecular identification revealed that the fungal strain was identified genetically as *Aspergillus niger* which is related to fungal strain *A. niger* strain AL-27 KC341933.1 that was deposited in the NCBI database with similarity percentages of 99%. The gene bank recorded the fungal strain *A. niger*, which was identified in the current study, under the accession number PV017292.

In a previous study, intestinal fungi particularly *Aspergillus* spp isolated from Three Species of Coral Reef Fish, where these fungi included *Aspergillus niger*,* A. medius*, *A. aculeatinus*, *A. ochraceopetaliformis*,* A. pseudoglaucus*,* A. restrictus* and *A. sydowii*^48^. Likewise, Long, Wu^49^ isolated *Aspergillus* from intestine of Black Carp and Grass Carp. Also, Ekanem, Itah^50^ reported that, isolated *Aspergillus* and *Penicillium* from fish gut of fish species (*Pseudotolithus typus* and *Chrysichthys nigrodigitatus*) from Qua Iboe River Estuary.

### GC-MS analysis

**Fig. 2:**
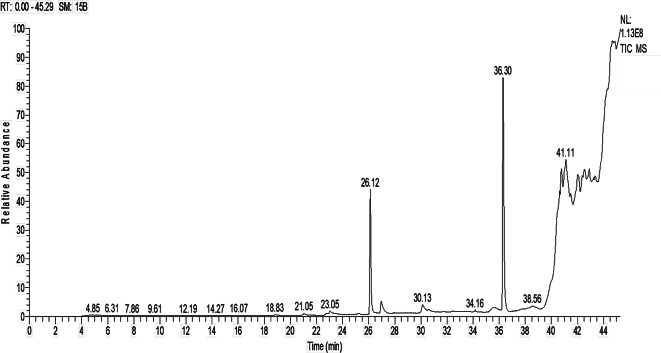
GC-MS chromatogram of EA of *A. niger*.

GC-MS is a potent analytical technique that is extensively employed to separate and identify volatile and semi-volatile compounds in a variety of samples^41,51^. In the current study, GC-MS was applied for fungal extract of *A. niger* to detect its compounds as shown in (Fig. 2; Table 1). Table 1 shows presence 8 compounds in the ethyl acetate extract of *A. niger* using GC-MS analysis. The most abundant compounds identified were Diisooctyl phthalate and 1,2-benzen dicaboxylic acid, bis (2-methoxyethyl) ester with percentages 54.32 and 26.32% respectively. Moreover, other low-percentage compounds found were hexadecanoic acid (5.43%), oleic acid (4.84%), 9-octadecanoic acid (Z)- (2.20%), Tetradecanoic acid (2.18%) and 2,2-dideutero octadecanal (0.98%). El-Enain et al.^52^ reported that, diisooctyl phthalate was the dominant and exhibited promising antimicrobial activity. Likewise, Al-Askar et al.^53^ confirmed that, diisooctyl phthalate is powerful bioactive compound where it showed potential antifungal activity. Furthermore, the bioactive compounds identified 1,2-benzenedicarboxylic acid and hexadecanoic acid are consistent with the research conducted by Siddiquee et al.^54^. Moreover, Lukitaningsih and Rumiyati^55^ reported that hexadecanoic acid has antioxidant, anti-inflammatory, and antimicrobial activities. Furthermore, tetradecanoic acid has Antimicrobial, antioxidant, nematicidal, anticancer activities^55,56^. Moreover, 9-octadecanoic acid (Z)- showed antibacterial and antioxidant activity^57,58^.

**Table 1:** GC-MS of the EA extract of *A. niger*.

Compound	RT	Peak area%	Mwt	MF	Activity	Reference
Tetradecanoic acid	23.04	2.18	288	C14H28O2	Antimicrobial, antioxidant, nematicidal, anticancer activities	^55,56^
1,2-benzen dicaboxylic acid, bis (2-methoxyethyl) ester	26.12	26.32	278	C16H22O4	Antibacterial activity	^59^
Hexadecanoic acid	26.96	5.43	256	C16H32O2	Antioxidant, antimicrobial, anti-inflammatory activity	^55^
Oleic acid	30.12	4.84	282	C18H34O2	Anti-inflammatory, antifungal, antibacterial, antioxidant activity	^60–62^
5-hydroxy-2,2-dimethyl-5,6 -bis-(2-oxopropyl)- cyclohexanone	35.54	1.61	254	C14H22O4		
Diisooctyl phthalate	36.30	54.32	390	C24H38O4	Antimicrobial, scavenging capacity	^52,53^
9-octadecanoic acid (Z)-	38.57	2.20	258	C16H34S	Antibacterial and antioxidant activity	^57,58^
2,2-dideutero octadecanal	39.91	0.98	270	C18H34D2O	Antimicrobial activity	^63,64^

### Phenolic compounds in EA extract of *A. niger*

**Fig. 3:**
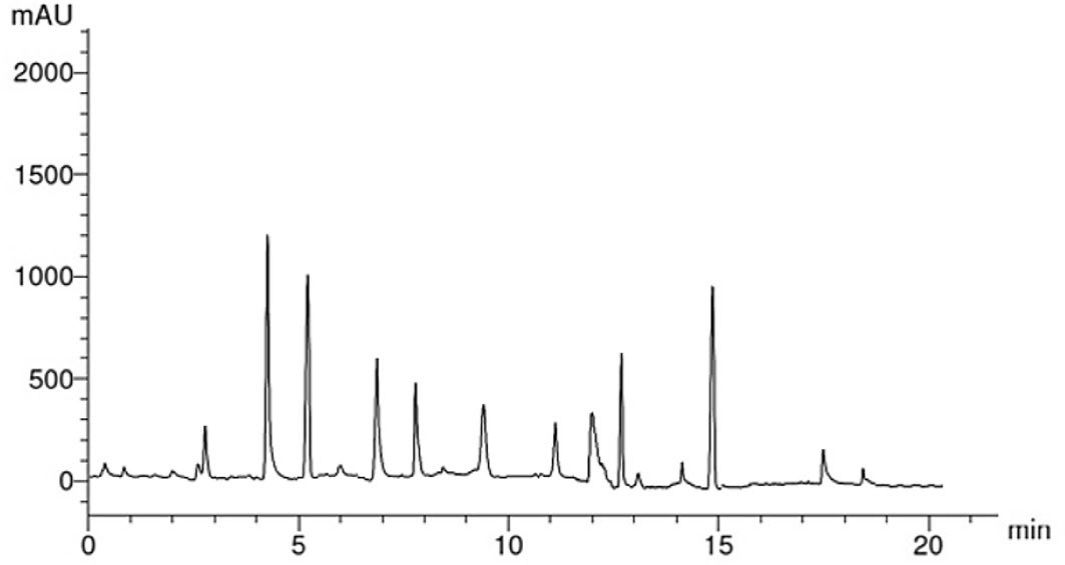
HPLC chromatogram of phenolic compounds identified in ethyl acetate extract of *A. niger.*

Phenolic compounds have different biological activities, making them beneficial for reducing oxidative stress and preventing chronic diseases^65^. Thus, quantities of phenolic compounds in EA extract of *A. niger* were determined using HPLC as shown in (Fig. 3). Results revealed that, the most abundant phenolic compounds in EA extract of *A. niger* were catechol (15.41 µg/mL), syringenic (13.25 µg/mL) as shown in (Table 2). Also, some of phenolic compounds were detected, cinnamic, caffeic, salicylic, pyrogallol, Chlorogenic and ferulic acid with percentages 7.41, 5.38, 4.65, 3.19, 2.55 and 2.49 µg/mL respectively. Surana et al.^66^ reported that catechol framework plays a crucial role in medicinal chemistry as it is found in numerous naturally occurring compounds with diverse biological activities. Furthermore, Caffeic acid has antimicrobial and antioxidant activities^67^. Feriotto et al.^68^ found that caffeic acid exhibits both anti-inflammatory and anticancer properties, specifically targeting certain leukemia cell lines. This suggests potential therapeutic applications for caffeic acid in treating leukemia by reducing inflammation and inhibiting cancer cell proliferation. Other researchers demonstrated that salicylic acid is a natural and safe antimicrobial agent^69^. Bio-functions of chlorogenic acid were reported, including antioxidant, anti-bacterial, anti-tumor, and anti-inflammatory, besides other therapeutic properties^70^. Ferulic acid was detected in *Aspergillus* sp. and exhibited antioxidant and antifungal activity^71^.

**Table 2:** Phenolic compounds of EA extract of *A. niger* by HPLC.

RT	Compound	Concentration (µg/mL)
2.9	Chlorogenic	2.55
4.0	Catechol	15.41
5.0	Syringenic	13.25
7.0	Cinnamic	7.41
8.0	Caffeic	5.38
9.0	Pyrogallol	3.19
11.0	Ferulic	2.49
12.0	salicylic	4.65

### Antimicrobial activity

**Fig. 4:**
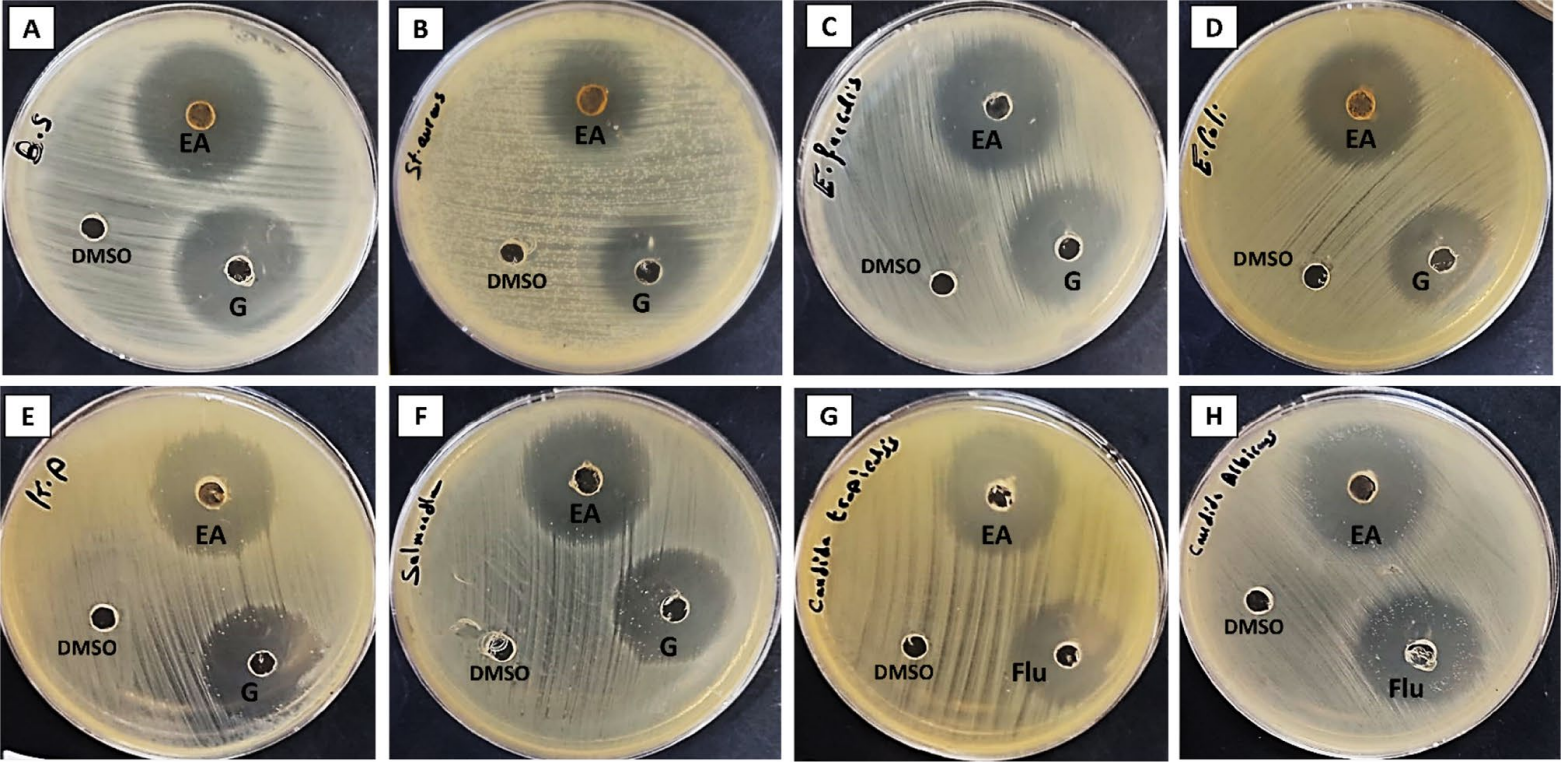
Antimicrobial activity of EA extract of *(A) niger* (EA), positive control (Gentamycin (G)/Fluconazole (Flu)), and DMSO against *(B) subtilis* (**A**), *S. aureus* (**B**), *E. faecalis* (**C**), *E. coli* (**D**), *K. pneumonia* (**E**), *S.typhimurium* (**F**), *(C) tropiclais* (**G**) and *C. albicans* (**H**).

Fungi represent a varied collection of life forms that are crucial to ecosystems, especially in the processes of nutrient cycling and decomposition^72^. Many fungi exhibit remarkable antimicrobial properties, generating a diverse range of bioactive substances that can inhibit the proliferation of bacteria, viruses, and other harmful microorganisms^73,74^. The potential of these antimicrobial properties is especially significant given the rising issue of antibiotic resistance, with marine fungi presenting an encouraging avenue for discovering new antimicrobial agents. In the current work, the antibacterial efficacy of EA extract of *A. niger* was carried out as shown in (Fig. 4). Results revealed that, EA extract of *A. niger* showed promising antibacterial activity against all bacterial tested strains compared to gentamicin. The most significant growth inhibition observed was 32 ± 0.1 mm in *B. subtilis*, with *E. faecalis* following closely at 30 ± 0.2 mm. The least growth inhibition observed was 22 ± 0.1 mm against *S. aureus*. Additionally, the extract from *A. niger* exhibited antifungal properties against *C. tropicalis* and *C. albicans*, showing inhibition zones of 28 ± 0.2 mm and 27 ± 0.2 mm, respectively (Table 3).

Liao et al.^48^ reported that *A. niger* isolated from the intestine of *Trachinotus blochii* fish exhibited high antibacterial activity. Additionally, they found other species of *Aspergillus* including *A. ochraceopetaliformis* and *A. pseudoglaucus* which are isolated from the intestine of *Lutjanus argentimaculatus*, and *Lates calcarifer* fish, respectively showed antibacterial activity against *vibrio alginolyticus* bacterium. Marine-derived *Aspergillus* sp. was isolated from the viscera of the barracuda and exhibited antibacterial effects against *B.subtilis*, from which four butyrolactones and methyl 2,4-dihydroxy-3,5,6-trimethylbenzoate compounds were identified. These compounds showed antibacterial effects against *S. aureus*,* Enterobacter aerogenes*,*B. subtilis*, and *E.coli*^75^. Previous studies suggested that the extraction via ethyl acetate exhibited the highest activity compared to others^76^. Similarly, research on fungal extracts demonstrated that EA extracts displayed the highest antimicrobial activity compared to those from methanol, water, and n-hexane^77,78^.

**Table 3:** Antimicrobial activity of EA crude extract of *A. niger* on tested bacterial and fungal strains.

Test microbial strains	Diameter inhibition zone in mm	
	*A. niger* EA extract	Gentamycin/fluconazole (1 mg/1 mL)
*B. subtilis*	32 ± 0.1	30 ± 0.2
*E. faecalis*	30 ± 0.2	26 ± 0.2
*S.aureus*	22 ± 0.1	24 ± 0.2
*E. coli*	27 ± 0.1	25 ± 0.1
*S. typhi*	28 ± 0.2	25 ± 0.2
*K. pneumoniae*	27 ± 0.2	24 ± 0.1
*C. tropicalis*	28 ± 0.2	22 ± 0.2
*C. albicans*	27 ± 0.2	27 ± 0.2

### Minimum inhibitory, bactericidal, and fungicidal activity of EA extract of *A. niger*

MIC and MBC assessments were conducted on all evaluated bacterial and fungal strains, as depicted in the (Table 4). The results indicated that the EA extract of *(A) niger* exhibited the lowest MIC of 7.8 µg/mL against *B. subtilis* and the lowest MBC of 15.62 µg/mL against *B. subtilis*,* K. pneumoniae*,* and C. tropicalis. E. coli* and *S. typhimurium* had the greatest MIC of 62.5 µg/mL, whereas *S. aureus* demonstrated the highest MBC of 500 µg/mL. The determined MBC/MIC index values demonstrated the bacteriostatic effect of *A. niger* extract on *S. aureus* and its bactericidal/fungicidal effect on other species. In a previous study, *Aspergillus sp.* From marine source exhibited antimicrobial activity toward MRSA, *E. faecalis*,* S. aureus*,* and K. pneumonia* where MIC values were 0.45–7.8 µg/mL^79^. Furthermore, the marine fungus *Aspergillus* sp.LS57 was shown to have antibacterial properties, with MIC values of 64 µg/mL against *S. aureus* and 128 µg/mL against *(B) subtilis* and *E. coli*^80,81^.

**Table 4:** MIC and MBC of EA extract of *A. niger* against tested pathogenic microbes.

Testes microbial strains	*A. niger* EA extract (µg/mL)		
	MIC	MBC/ MFC	(MBC or MFC)/MIC
*B. subtilis*	7.8	15.62	2
*E. faecalis*	31.25	62.5	2
*S. aureus*	125	500	4
*E. coli*	62.5	125	2
*S. typhi*	62.5	125	2
*K. pneumoniae*	15.62	15.62	1
*C. tropicalis*	7.8	15.62	2
*C. albicans*	31.25	62.5	2

### Anti-biofilm activity

Microbial biofilms are compact aggregates of bacteria that attach to surfaces and are encased in a self-generated extracellular matrix, rendering them resistant to environmental stresses and antimicrobial treatments^82^. One effective approach to destructing biofilms is the application of antimicrobial agents, which can penetrate the biofilm matrix and target the embedded microorganisms. Antimicrobial agents, such as antibiotics, disinfectants, and essential oils, are used to disrupt the biofilm’s integrity and inhibit microbial growth^83^. In this study, EA extract of *A. niger* was assessed for antibiofilm activity toward some of tested bacterial strains as shown in (Table 5). Results illustrated that, EA extract of *A. niger* showed promising antibiofilm activity toward *S. typhimurium* with percentages 94.49, 87.72, and 85.97% at 75, 50 and 25% of MIC respectively. Moreover, it exhibited antibiofilm activity toward *S. aureus*,* E. faecalis* and *E.coli* but less than *S. tphimurium* with percentages (90.36, 82.87, 32.85%), (87.81, 81.91, 66.98%), and (88.80, 76.82, 48.08%) at 75, 50 and 25% of MIC respectively (Table 5). In a previous study, *.A. niger* exhibits notable antibiofilm effectiveness, achieving inhibition rates of 95.3%, 74.9%, 77.1%, and 93.6% against *E.coli*,* P. aeruginosa*,* Proteus mirabilis*, and MRSA, respectively^84^. The findings align with the data presented by Hamed et al.^85^, which showed that an ethyl acetate crude extract from *Aspergillus* sp. SO12, sourced from marine environments, displayed significant biofilm inhibitory effects against *S. aureus*, *E. coli*, *B.* *subtilis*, and *P. aeruginosa*, achieving percentages of 70.23%, 45.25%, 35.23%, and 20.02%, respectively. Also, several MDR bacteria were shown to have their biofilm production inhibited by Aspulvinones B, H, R, and S, which are produced from the marine fungus *Aspergillus* sp^81,86^.

### The effect of EA extract of *(A) niger* on *(B) subtilis* under TEM

**Fig. 5:**
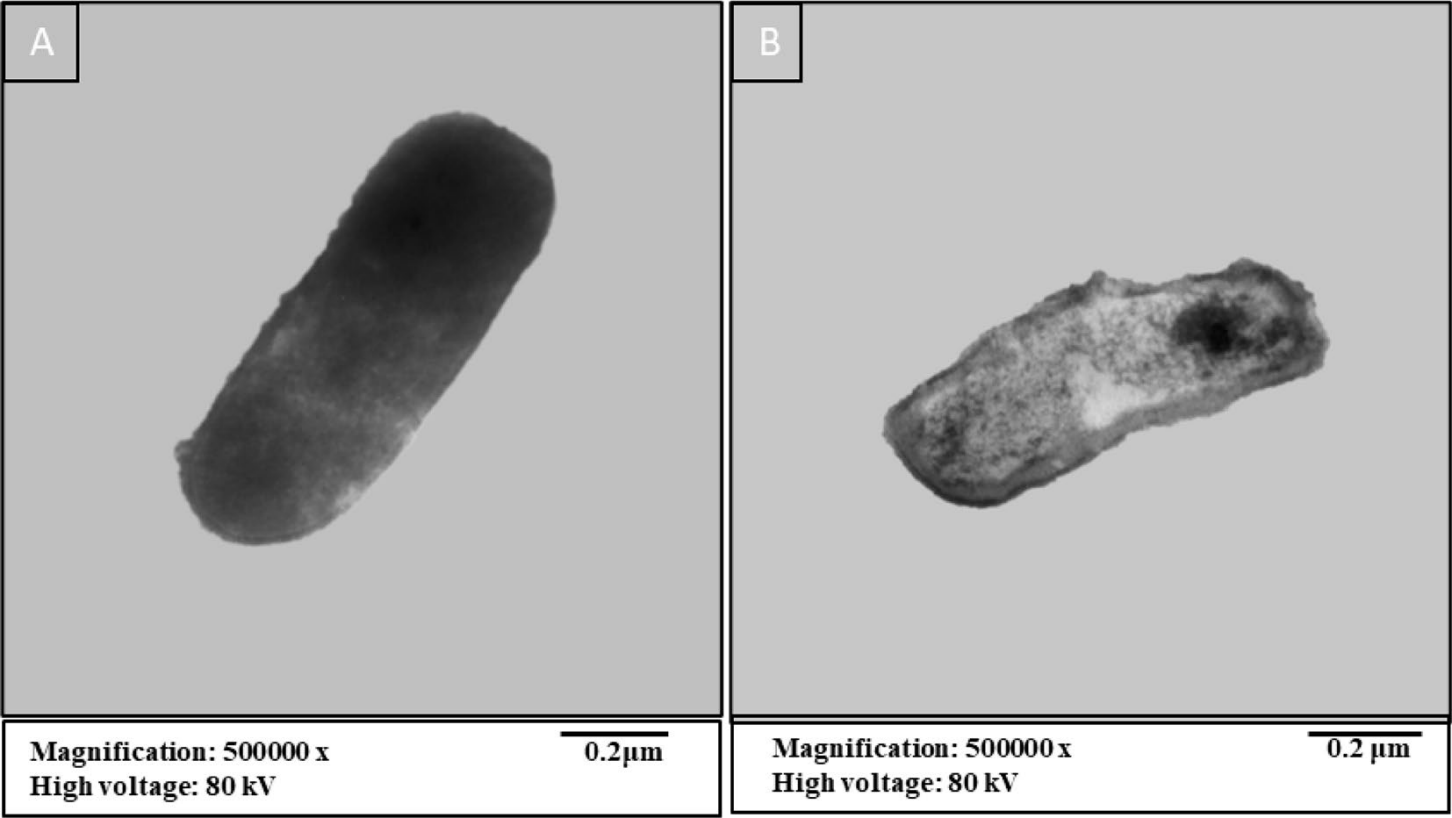
Transmission electronic micrographs of *B.subtilis*. (**A**) control (untreated) and (**B**) treated cell with EA extract of *A. niger*.

To confirm the antibacterial activity of EA extract of *(A) niger*, TEM was employed to examine the ultrastructure of *Bacillus subtilis* treated with this extract as illustrated in (Fig. 5). The transmission electron micrograph of typical *(B) subtilis* reveals rod-shaped cells characterized by a smooth, continuous cell wall and an intact cell membrane. The cytoplasm appears homogeneous and electron-dense, indicating robust cellular integrity. Additionally, a normal electron-lucent zone is observed between the cell wall and the cell membrane, which is indicative of healthy cellular structure. These features highlight the typical morphology of *B. subtilis*, providing a baseline for comparison with treated cells, especially in studies assessing the effects of antibacterial agents (Fig. 5A). In contrast, *B. subtilis* treated with EA extract of *A. niger* exhibited significant alterations in cellular structure, as evidenced by transmission electron microscopy. The treated cells showed an enlarged periplasmic region between the outer membrane and the cytoplasmic membrane, indicating disruption of the normal cellular architecture. Severe damage was evident in lysed cells, which displayed a disintegrated cell wall and ruptured cytoplasmic membrane, leading to the leakage of cytoplasmic contents. These destructive changes underscore the antibacterial effectiveness of the EA extract, highlighting its potential to compromise bacterial cell integrity, as illustrated in (Fig. 5B). According to Yassein, Hassan^84^ the crude extract of *A. niger* induced distinct morphological changes in various tested bacteria. In *E. coli*, the cells became shorter and smaller, indicating a reduction in size and possibly viability. For *Proteus mirabilis*, the cells exhibited a curved shape and showed signs of division, suggesting alterations in their growth dynamics. In the case of *P. aeruginosa*, the cells were distorted, transitioning from a bacillus shape to a more spherical form, which reflects significant morphological disruption. Finally, MRSA cells began to swell and adopt an irregular spherical shape, indicating severe cellular stress and potential loss of structural integrity.

**Table 5:** Antibiofilm activity of EA extract of *A. niger* against tested pathogenic bacteria.

Pathogenic strains	Antibiofilm activity (%)		
	75% of MIC	50% of MIC	25% of MIC
*S. typhimurium*	94.49 ± 0.37	87.72 ± 0.51	85.97 ± 0.36
*S. aureus*	90.36 ± 0.35	82.87 ± 0.55	32.85 ± 0.27
*E. faecalis*	87.81 ± 0.39	81.91 ± 0.59	66.98 ± 0.15
*E. coli*	88.80 ± 0.47	76.82 ± 0.58	48.08 ± 0.34

### Antioxidant activity of fungal extract

**Fig. 6:**
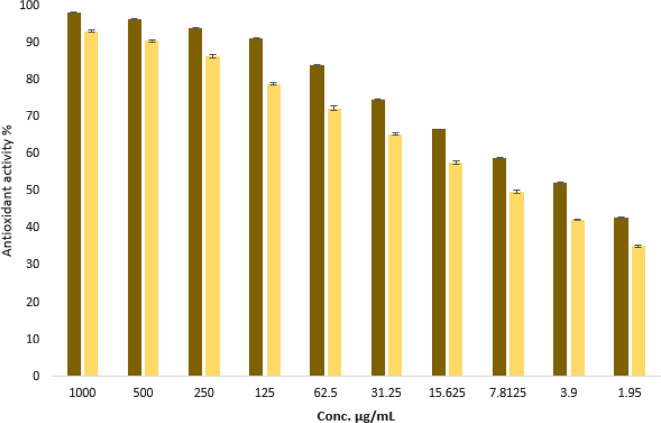
Antioxidant activity of EA extract of *A. niger* using DPPH method.

Compounds that act as antioxidants are essential for protecting cells from oxidative stress by countering harmful free radicals, which can damage critical cellular components such as DNA, proteins, and lipids. Antioxidants reduce the risk of chronic diseases, such as cancer, cardiovascular issues, and neurodegenerative disorders, by scavenging and neutralizing these free radicals, which in turn mitigates oxidative damage. This protective function emphasizes the significance of antioxidants in the preservation of cellular health and the enhancement of overall well-being^87,88^. In this study, the antioxidant activity of the ethyl acetate (EA) extract of *A. niger* was assessed at different concentrations using the DPPH method. The findings demonstrated that the EA extract showed notable antioxidant activity, presenting an IC50 value of 8.17 µg/mL, in contrast to ascorbic acid (AA), which recorded an IC50 of 2.97 µg/mL (Fig. 6). FRAP method was also used to evaluate antioxidant activity of the *A. niger* extract. Results revealed that, *A. niger* extract exhibited antioxidant activity with ascorbic acid equivalent (AAE) 732.5 ± 3.4 µg/mg. Yang et al.^89^ observed that marine *A. versicolor* SH0105 exhibited powerful reduction of Fe^3+^ with the FRAP value of 9.0 mM under the concentration of 3.1 µg/mL, which was more potent than ascorbic acid. In prior research, they found that *A. niger* extract is high in phenolic compounds and has strong antioxidant activity in vitro^90^. *Aspergillus* sp. isolated from Barracuda fish exhibited good scavenging activity with an EC_50_ value of 2.8 mg/mL against DPPH free radicals^91^. Also, rubrolide R a natural product derived from *Aspergillus* sp. which isolated from the viscera of *chelon haematocheilus* fish exhibited antioxidant activity with IC50 1.3 Mm^75^. The findings robustly endorse the utilization of ethyl acetate crude extract of *A. niger* as an effective natural antioxidant for health maintenance against various oxidative stress linked to degenerative diseases.

### Computational procedures

**Fig. 7:**
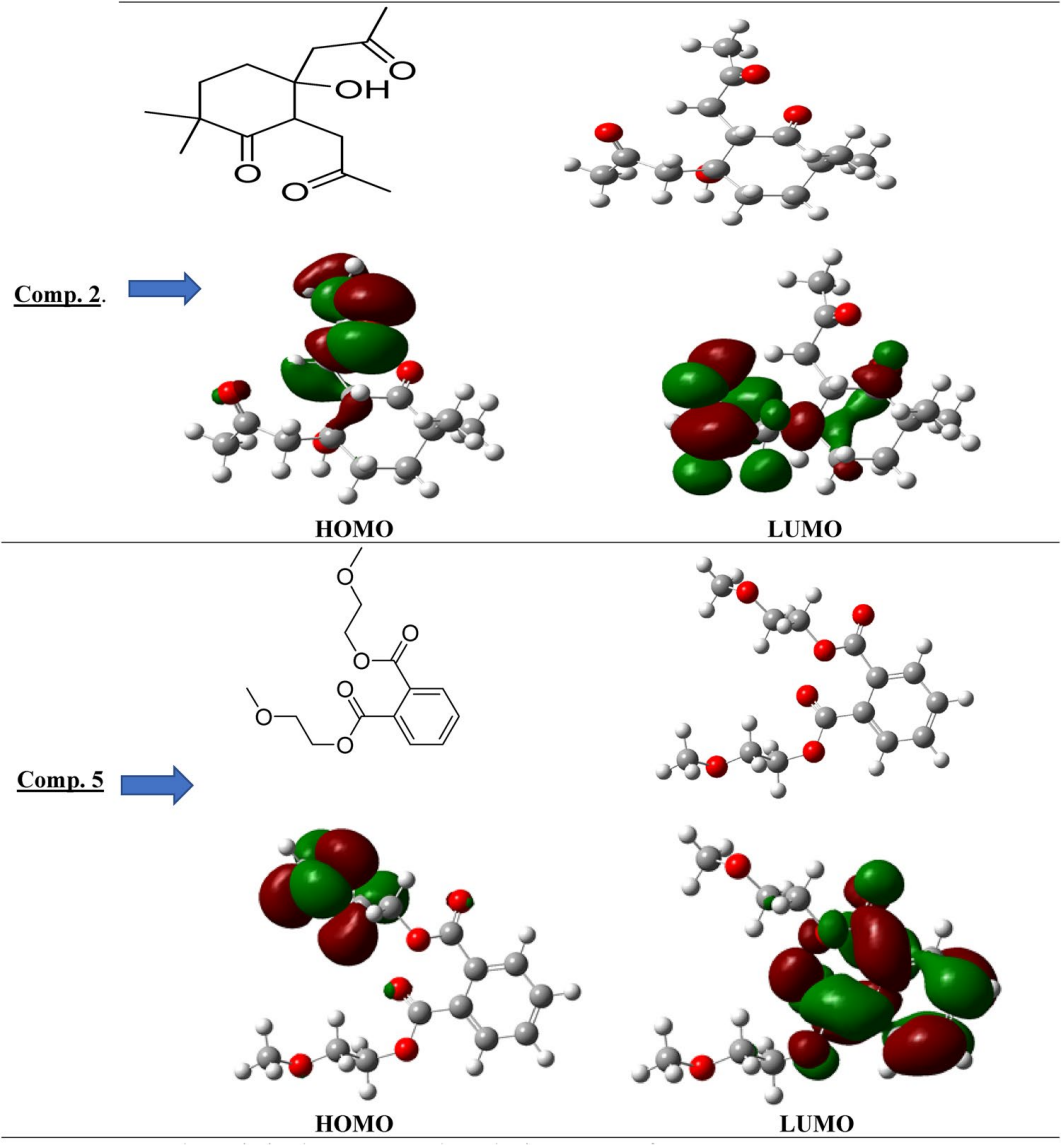
The optimized geometry and numbering system of comp. 2 & 5 and Gap energy (HOMO–LUMO).

DFT calculations are a valuable method employed to investigate the reactivity of chemicals. The optimized geometries of 1,2-benzenedicarboxylic acid, bis(2-methoxyethyl) ester (compound 2), and 5-hydroxy-2,2-dimethyl-5,6-bis(2-oxopropyl)cyclohexanone (compound 5) were computed using DFT B3LYP/6-31G(d) methodology. Table 6 presents metrics that indicate the reactivity and stability of molecules, including total energy (ET), energy of the highest occupied molecular orbital (EHOMO), energy of the lowest unoccupied molecular orbital (ELUMO), and the energy gap (Eg). Figure 7 illustrates the molecular structure of compounds 2 and 5, together with the reactivity of the isolated compounds. As the energy gap (Eg) diminishes, it facilitates the passage of electrons from lower orbitals to higher ones, hence enhancing the likelihood of reactions in the compounds^92,93^.

**Table 6:** Optimized geometries of (comp. 2 & 5).

Parameter	Comp. 2	Comp. 5
	DFT 3LYP/6-311G (d)	DFT 3LYP/6-311G (d)
ET (au)	−847.390	−995.3505
EHOMO(eV)	−0.23556	−0.2497
ELUMO (ev)	−0.03979	−0.06816
ΔE (ev)	0.19577	0.18154
µ (Debye)	6.3034	3.2325

$$\Delta {\text{E = ELUMO}} - {\text{EHOMO}}$$

### Molecular modeling and docking

**Fig. 8:**
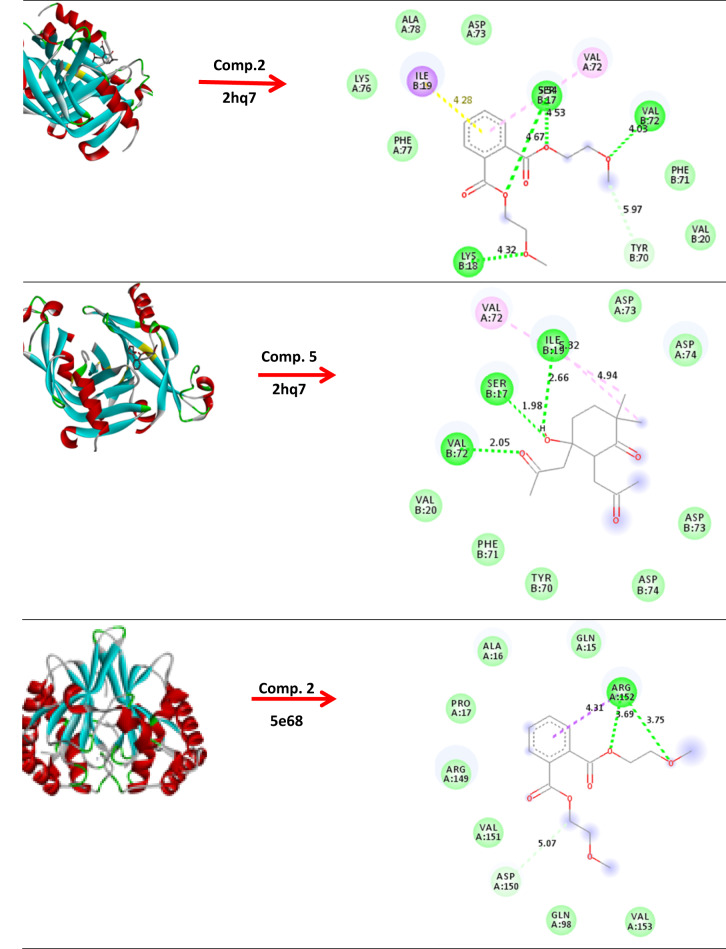

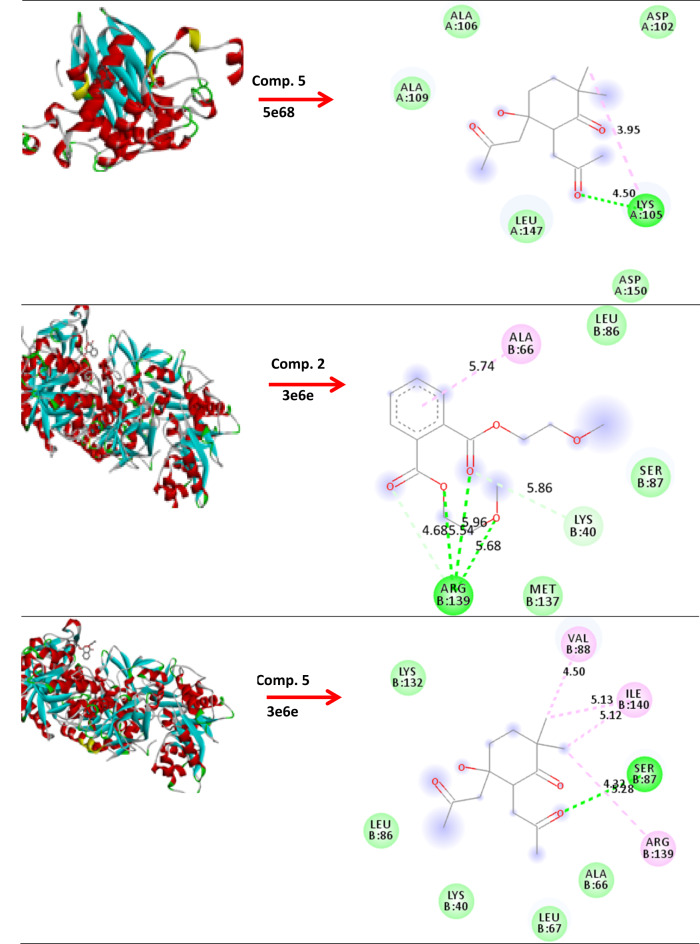

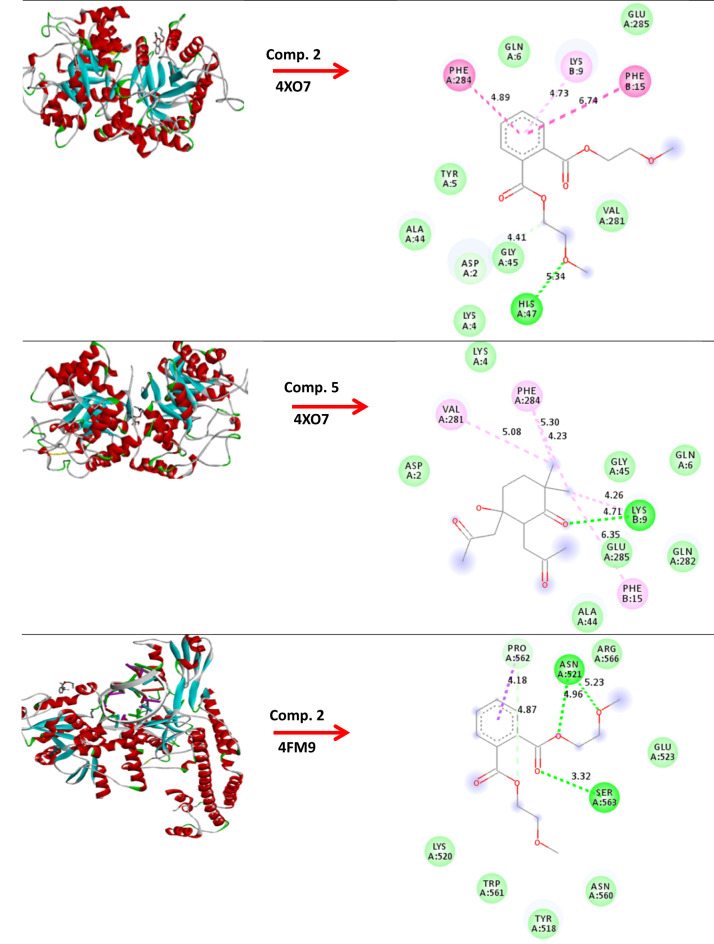

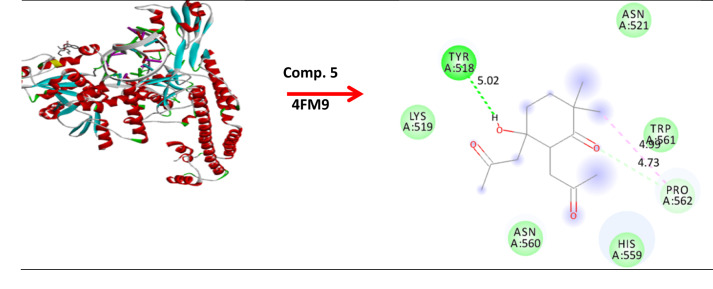
Molecular docking of comp. 2 & 5 with different protein.

The growth inhibition of pathogenic microorganisms by EA crude extracts of *(A) niger* has been examined. The molecular modeling of *(B) subtilis* (ATCC 6633) (PDB: 2hq7) crystal structure of the LuxS-Quorum sensor molecular complex from Salmonella typhi, *S. aureus* (ATCC 6538) (PDB: 3e6e), and the crystal structure of the Protein related to general stress protein 26 (GS26) of *B. subtilis* (pyridoxinephosphate oxidase family), *E. faecalis* (ATCC 29212) (PDB: 5e68) (Fig. 8). The crystal structure of Alanine racemase from *E. faecalis* in complex with cycloserine, with prediction of anticancer activity of breast cancer MCF-7 (PDB: 4XO7) and hepatic HepG2(PDB: 4FM9) as a protein receptor against comp. 2 & 5 as a ligand has studied via molecular docking investigation with good bond lengths and energy as in (Table 7). As the bond length decease the ligand became closer to the receptor and more reactive. A perfect bond length reaches 0.8 Å and 1.0 Å in case breast cancer MCF-7 (PDB: 4XO7) and hepatic HepG2, so we expect the compounds have anticancer activity^94^.

**Table 7:** Molecular docking of comp. 2 & 5 with bond lengths and energy.

Protein( PDB: ID )	Comp. 2	Comp. 5
2hq7	(−5.3 1.518Å)	(−5.4 1.695Å)
5e68	(−3.5 1.755 Å)	(−3.6 1.572Å)
3e6e	(−4.8 1.837 Å)	(−4.0 2.143 Å)
4XO7	(−4.5 1.618 Å)	(−5.4 0.841 Å)
4FM9	(−4.1 1.316 Å)	(−4.0 1.001 Å)

## Conclusion

In the current study, *A. niger* was isolated from the gut of *Scarus ghobban* for the first time. The ethyl acetate extract of *A. niger* has significant antibacterial, antibiofilm, and antioxidant characteristics, making it a suitable option for the development of new antimicrobial agents. The identification of essential bioactive components using GC-MS and HPLC investigations emphasizes its medicinal potential. The extract’s efficacy against both Gram-positive and Gram-negative bacteria, together with notable antifungal properties, underscores its adaptability in addressing various diseases. Additionally, the observed potent antioxidant activity suggests that *A. niger* could play a crucial role in addressing oxidative stress-related conditions. These findings not only contribute to the growing body of research on fungal extracts but also pave the way for future studies aimed at harnessing *A. niger* for innovative applications in pharmaceuticals and biotechnology. DFT calculation and molecular docking showed good compounds reactivity and good prediction for cancer cell with breast cancer MCF-7 (PDB: 4XO7) and hepatic HepG2 with short bond length.

## Data Availability

The datasets analyzed during the current study are available in the NCBI GenBank database repository with the accession number of PV017292, https://www.ncbi.nlm.nih.gov/nuccore/PV017292.

## Declarations

### **Accordance and arrive guidelines statement**

*Accordance*: We confirmed that all experiments in this study were performed in accordance with the relevant guidelines and regulations. *Arrive*: All the procedure of the study is followed by the ARRIVE guidelines.

## References

[1] Shi, X. et al. Challenges of point-of-use devices in purifying tap water: the growth of biofilm on filters and the formation of disinfection byproducts. *Chem. Eng. J.* **462**, 142235 (2023).

[2] Hage, M. et al. Cold plasma surface treatments to prevent biofilm formation in food industries and medical sectors. *Appl. Microbiol. Biotechnol.* 1–20 (2022). 10.1007/s00253-021-11715-y PMC8661349 34889984

[3] Xu, W. & Koydemir, H. C. Non-invasive biomedical sensors for early detection and monitoring of bacterial biofilm growth at the point of care. *Lab. Chip*. **22** (24), 4758–4773 (2022). 36398687 10.1039/d2lc00776b

[4] Nudelman, R. et al. Bio-assisted synthesis of bimetallic nanoparticles featuring antibacterial and photothermal properties for the removal of biofilms. *J. Nanobiotechnol.* **19**, 1–10 (2021). 10.1186/s12951-021-01183-x PMC8715638 34963478

[5] Mevo, S. I. U. et al. Promising strategies to control persistent enemies: some new technologies to combat biofilm in the food industry—A review. *Compr. Rev. Food Sci. Food Saf.* **20** (6), 5938–5964 (2021). 34626152 10.1111/1541-4337.12852

[6] Gopalakrishnan, S. et al. Ultrasound-enhanced antibacterial activity of polymeric nanoparticles for eradicating bacterial biofilms. *Adv. Healthc. Mater.* **11** (21), 2201060 (2022). 10.1002/adhm.202201060 PMC9633556 36049222

[7] Fisher, R. et al. Species richness on coral reefs and the pursuit of convergent global estimates. *Curr. Biol.* **25** (4), 500–505 (2015). 25639239 10.1016/j.cub.2014.12.022

[8] Brandl, S. J., Casey, J. M. & Meyer, C. P. Dietary and habitat niche partitioning in congeneric cryptobenthic reef fish species. *Coral Reefs*. **39** (2), 305–317 (2020).

[9] Ringø, E. et al. Effect of dietary components on the gut microbiota of aquatic animals. A never-ending story? *Aquacult. Nutr.* **22** (2), 219–282 (2016).

[10] Foster, K. R. et al. The evolution of the host Microbiome as an ecosystem on a leash. *Nature* **548** (7665), 43–51 (2017). 28770836 10.1038/nature23292 PMC5749636

[11] Ganguly, S. & Prasad, A. Microflora in fish digestive tract plays significant role in digestion and metabolism. *Rev. Fish Biol. Fish.* **22**, 11–16 (2012).

[12] Rooks, M. G. & Garrett, W. S. Gut microbiota, metabolites and host immunity. *Nat. Rev. Immunol.* **16** (6), 341–352 (2016). 27231050 10.1038/nri.2016.42 PMC5541232

[13] Talwar, C. et al. Fish gut microbiome: current approaches and future perspectives. *Indian J. Microbiol.* **58**, 397–414 (2018). 30262950 10.1007/s12088-018-0760-y PMC6141390

[14] Dell’Anno, F. et al. Fungi can be more effective than bacteria for the bioremediation of marine sediments highly contaminated with heavy metals. *Microorganisms* **10** (5), 993 (2022). 35630436 10.3390/microorganisms10050993 PMC9145406

[15] Liu, Z. et al. Fungi: outstanding source of novel chemical scaffolds. *J. Asian Nat. Prod. Res.* **22** (2), 99–120 (2020). 30047298 10.1080/10286020.2018.1488833

[16] Li, K. et al. Natural products from Mangrove sediments-derived microbes: structural diversity, bioactivities, biosynthesis, and total synthesis. *Eur. J. Med. Chem.* **230**, 114117 (2022). 35063731 10.1016/j.ejmech.2022.114117

[17] Jiang, M. et al. A review of terpenes from marine-derived fungi: 2015–2019. *Mar. Drugs*. **18** (6), 321 (2020). 32570903 10.3390/md18060321 PMC7345631

[18] Qadri, H. et al. Natural products and their semi-synthetic derivatives against antimicrobial-resistant human pathogenic bacteria and fungi. *Saudi J. Biol. Sci.* **29** (9), 103376 (2022). 35874656 10.1016/j.sjbs.2022.103376 PMC9290337

[19] Qi, J. et al. Genomic analysis and antimicrobial components of M7, an Aspergillus terreus strain derived from the South China sea. *J. Fungi*. **8** (10), 1051 (2022). 10.3390/jof8101051 PMC9605573 36294615

[20] Mia, M. M. et al. Inhibitory potentiality of secondary metabolites extracted from marine fungus target on avian influenza virus-a subtype H5N8 (Neuraminidase) and H5N1 (nucleoprotein): a rational virtual screening. *Veterinary Anim. Sci.* **15**, 100231 (2022). 10.1016/j.vas.2022.100231 PMC8760399 35059528

[21] Zhao, H. et al. Investigation of the bactericidal mechanism of penicilazaphilone C on Escherichia coli based on 4D label-free quantitative proteomic analysis. *Eur. J. Pharm. Sci.* **179**, 106299 (2022). 36179970 10.1016/j.ejps.2022.106299

[22] Xu, X. et al. Investigation on the chemical constituents of the marine-derived fungus strain Aspergillus brunneoviolaceus MF180246. *Nat. Prod. Res.* **38** (8), 1369–1374 (2024). 36369790 10.1080/14786419.2022.2144300

[23] Moglad, E. et al. Antibacterial and anti-Toxoplasma activities of Aspergillus Niger endophytic fungus isolated from Ficus retusa: in vitro and in vivo approach. *Artif. Cells Nanomed. Biotechnol.* **51** (1), 297–308 (2023). 37224186 10.1080/21691401.2023.2215531

[24] Rasouli, R. et al. Antibiofilm activity of cellobiose dehydrogenase enzyme (CDH) isolated from Aspergillus Niger on biofilm of clinical Staphylococcus epidermidis and Pseudomonas aeruginosa isolates. *Arch. Clin. Infect. Dis.* **15** (1) (2020).

[25] Xu, S. et al. Analysis of gut-associated fungi from Chinese mitten crab Eriocheir sinensis. *All Life* **14** (1), 610–621 (2021).

[26] Tamura, K., Stecher, G. & Kumar, S. MEGA11: molecular evolutionary genetics analysis version 11. *Mol. Biol. Evol.* **38** (7), 3022–3027 (2021). 33892491 10.1093/molbev/msab120 PMC8233496

[27] Goz, R. A. E. et al. Isolation of some toxigenic fungi from sugarcane juice. *J. Basic. Environ. Sci.* **11** (4), 341–360 (2024).

[28] Sharaf, M. H. et al. Antimicrobial, antioxidant, cytotoxic activities and phytochemical analysis of fungal endophytes isolated from ocimum basilicum. *Appl. Biochem. Biotechnol.* 1–19 (2022). 10.1007/s12010-021-03702-w 34661866

[29] Fayed, M. A., Abouelela, M. E. & Refaey, M. S. Heliotropium ramosissimum metabolic profiling, in Silico and in vitro evaluation with potent selective cytotoxicity against colorectal carcinoma. *Sci. Rep.* **12** (1), 12539 (2022). 35869153 10.1038/s41598-022-16552-1 PMC9307647

[30] Bakr, R. O. et al. In-vivo wound healing activity of a novel composite sponge loaded with mucilage and lipoidal matter of hibiscus species. *Biomed. Pharmacother.* **135**, 111225 (2021). 10.1016/j.biopha.2021.111225 33434856

[31] Kuntić, V. et al. Isocratic RP-HPLC method for Rutin determination in solid oral dosage forms. *J. Pharm. Biomed. Anal.* **43** (2), 718–721 (2007). 16920326 10.1016/j.jpba.2006.07.019

[32] Rabie, M. et al. Transcriptional responses and secondary metabolites variation of tomato plant in response to tobacco mosaic virus infestation. *Sci. Rep.* **14** (1), 19565 (2024). 39174617 10.1038/s41598-024-69492-3 PMC11341961

[33] PA, W. Reference method for broth dilution antifungal susceptibility testing of yeasts, approved standard. CLSI document M27-A2, (2002).

[34] Hashem, A. H. & El-Sayyad, G. S. Antimicrobial and anticancer activities of biosynthesized bimetallic silver-zinc oxide nanoparticles (Ag-ZnO NPs) using pomegranate Peel extract. *Biomass Convers. Biorefinery*. **14** (17), 20345–20357 (2024).

[35] Elsayed, Y. M. et al. Antibacterial activity of ethanolic extracts of Thymus vulgaris and Cinnamomum camphora on human pathogenic bacteria. *J. Basic. Environ. Sci.* **11** (4), 437–452 (2024).

[36] Shehabeldine, A. M. et al. Potential antimicrobial and antibiofilm properties of copper oxide nanoparticles: time-kill kinetic essay and ultrastructure of pathogenic bacterial cells. *Appl. Biochem. Biotechnol.* **195** (1), 467–485 (2023). 36087233 10.1007/s12010-022-04120-2 PMC9832084

[37] Elkady, F. M. et al. Unveiling the Launaea nudicaulis (L.) Hook medicinal bioactivities: phytochemical analysis, antibacterial, antibiofilm, and anticancer activities. *Front. Microbiol.* **15**, 1454623 (2024). 39421554 10.3389/fmicb.2024.1454623 PMC11484093

[38] Al-Rajhi, A. M. et al. The green approach of chitosan/Fe2O3/ZnO-nanocomposite synthesis with an evaluation of its biological activities. *Appl. Biol. Chem.* **67** (1), 75 (2024).

[39] Antunes, A. L. S. et al. Application of a feasible method for determination of biofilm antimicrobial susceptibility in Staphylococci. *Apmis* **118** (11), 873–877 (2010). 20955460 10.1111/j.1600-0463.2010.02681.x

[40] Amin, B. H. et al. Synthesis, characterization, and biological investigation of new mixed-ligand complexes. *Appl. Organomet. Chem.* **34** (8), e5689 (2020).

[41] Elghaffar, R. Y. A. et al. Promising endophytic Alternaria alternata from leaves of Ziziphus spina-christi: phytochemical analyses, antimicrobial and antioxidant activities. *Appl. Biochem. Biotechnol.* **194** (9), 3984–4001 (2022). 35579741 10.1007/s12010-022-03959-9 PMC9424163

[42] Salem, S. S. et al. Pseudomonas indica-mediated silver nanoparticles: antifungal and antioxidant biogenic tool for suppressing mucormycosis fungi. *J. Fungi*. **8** (2), 126 (2022). 10.3390/jof8020126 PMC8874487 35205879

[43] Abd Elghaffar, R. Y. et al. The potential biological activities of Aspergillus luchuensis-aided green synthesis of silver nanoparticles. *Front. Microbiol.* **15**, 1381302 (2024). 38832112 10.3389/fmicb.2024.1381302 PMC11146671

[44] Benzie, I. F. & Strain, J. J. The ferric reducing ability of plasma (FRAP) as a measure of antioxidant power: the FRAP assay. *Anal. Biochem.* **239** (1), 70–76 (1996). 8660627 10.1006/abio.1996.0292

[45] Athamena, S. et al. The antioxidant, anti-inflammatory, analgesic and antipyretic activities of Juniperu thurifera. *J. Herbs Spices Med. Plants*. **25** (3), 271–286 (2019).

[46] Fahim, A. M. et al. Antimicrobial, anticancer activities, molecular docking, and DFT/B3LYP/LANL2DZ analysis of heterocyclic cellulose derivative and their Cu-complexes. *Int. J. Biol. Macromol.* **269**, 132027 (2024). 38702001 10.1016/j.ijbiomac.2024.132027

[47] Elsayed, G. H., Dacrory, S. & Fahim, A. M. Anti-proliferative action, molecular investigation and computational studies of novel fused heterocyclic cellulosic compounds on human cancer cells. *Int. J. Biol. Macromol.* **222**, 3077–3099 (2022). 36244535 10.1016/j.ijbiomac.2022.10.083

[48] Liao, X. et al. Diversity and antimicrobial activity of intestinal fungi from three species of coral reef fish. *J. Fungi*. **9** (6), 613 (2023). 10.3390/jof9060613 PMC10299458 37367549

[49] Long, C. X. et al. Association of fungi in the intestine of black carp and grass carp compared with their cultured water. *Aquac. Res.* **2023** (1), 5553966 (2023).

[50] Ekanem, J. O., Itah, A. Y. & Ndubuisi-Nnaji, U. Microbial diversity, heavy metals and hydrocarbons concentration in some fish species from qua Iboe river estuary, Akwa Ibom state, Nigeria. *GSC Adv. Res. Reviews*. **16** (1), 242–251 (2023).

[51] Guo, L. et al. Simultaneous determination of five synthetic antioxidants in edible vegetable oil by GC–MS. *Anal. Bioanal. Chem.* **386**, 1881–1887 (2006). 16972057 10.1007/s00216-006-0738-1

[52] El-Enain, A. et al. Diisooctyl phthalate as A secondary metabolite from actinomycete inhabit animal’s Dung with promising antimicrobial activity. *Egypt. J. Chem.* **66** (12), 261–277 (2023).

[53] Al-Askar, A. et al. Diisooctyl phthalate, the major secondary metabolite of Bacillus subtilis, could be a potent antifungal agent against rhizoctonia solani: GC-MS and in Silico molecular Docking investigations. *Egypt. J. Chem.* **67** (13), 1137–1148 (2024).

[54] Siddiquee, S. et al. Separation and identification of hydrocarbons and other volatile compounds from cultures of Aspergillus Niger by GC–MS using two different capillary columns and solvents. *J. Saudi Chem. Soc.* **19** (3), 243–256 (2015).

[55] Lukitaningsih, E. & Rumiyati, R. GC-MS analysis of bioactive compounds in ethanol and Ethyl acetate fraction of grapefruit (Citrus maxima L.) rind. *Borneo J. Pharm.* **4** (1), 29–35 (2021).

[56] Ali, A. et al. Identification of the phytoconstituents in methanolic extract of Adhatoda vasica L. leaves by GC-MS analysis and its antioxidant activity. *J. AOAC Int.* **105** (1), 267–271 (2022). 34459903 10.1093/jaoacint/qsab113

[57] Qanash, H. et al. Anticancer, antioxidant, antiviral and antimicrobial activities of Kei Apple (Dovyalis caffra) fruit. *Sci. Rep.* **12** (1), 5914 (2022). 35396383 10.1038/s41598-022-09993-1 PMC8990652

[58] Sivakumar, S. GC-MS analysis and antibacterial potential of white crystalline solid from red algae portieria hornemannii against the plant pathogenic bacteria Xanthomnas axonopodis pv. citri (Hasse) Vauterin et al. and Xanthomonas campestris pv. malvacearum (smith 1901) dye 1978b. *Int. J. Adv. Res.* **2** (3), 174–183 (2014).

[59] Atallah, B. M., Haroun, S. A. & El-Mohsnawy, E. Antibacterial activity of two actinomycetes species isolated from black sand in North Egypt. *South Afr. J. Sci*. **119** (11–12), 1–8 (2023).

[60] Santa-María, C. et al. Update on anti-inflammatory molecular mechanisms induced by oleic acid. *Nutrients* **15** (1), 224 (2023). 36615882 10.3390/nu15010224 PMC9824542

[61] Ramadan, A. M. A. A. et al. Antioxidant, antibacterial, and molecular Docking of Methyl ferulate and oleic acid produced by Aspergillus pseudodeflectus AUMC 15761 utilizing wheat Bran. *Sci. Rep.* **14** (1), 3183 (2024). 38326360 10.1038/s41598-024-52045-z PMC10850474

[62] Momodu, I. et al. Gas chromatography–mass spectrometry identification of bioactive compounds in methanol and aqueous seed extracts of Azanza Garckeana fruits. *Nigerian J. Biotechnol.* **38** (1), 25–38 (2022).

[63] El-Fayoumy, E. A. et al. Evaluation of antioxidant and anticancer activity of crude extract and different fractions of Chlorella vulgaris axenic culture grown under various concentrations of copper ions. *BMC Complement. Med. Ther.* **21**, 1–16 (2021). 33546663 10.1186/s12906-020-03194-x PMC7863377

[64] Dawwam, G. E. et al. Analysis of different bioactive compounds conferring antimicrobial activity from Lactobacillus plantarum and Lactobacillus acidophilus with gas chromatography-mass spectrometry (GC-MS). *Egypt. Acad. J. Biol. Sci. G Microbiol.* **14** (1), 1–10 (2022).

[65] Sun, W. & Shahrajabian, M. H. Therapeutic potential of phenolic compounds in medicinal plants-natural health products for human health. **28** (4). (2023). 10.3390/molecules28041845 PMC9960276 36838831

[66] Surana, K. R. et al. Catechol: important scafold in medicinal chemistry. *MedicoPharmaceutica (MedicoPharm)* **1** (1), 47–57 (2023).

[67] Jokubaite, M. & Ramanauskiene, K. Potential unlocking of biological activity of caffeic acid by incorporation into hydrophilic gels. *Gels* **10** (12), 794 (2024). 39727552 10.3390/gels10120794 PMC11675749

[68] Feriotto, G. et al. Caffeic acid enhances the Anti-Leukemic effect of Imatinib on chronic myeloid leukemia cells and triggers apoptosis in cells sensitive and resistant to Imatinib. *Int. J. Mol. Sci.* **22** (4), 1644 (2021). 33562019 10.3390/ijms22041644 PMC7914550

[69] Song, X. et al. Antibacterial effect and possible mechanism of Salicylic acid microcapsules against *Escherichia coli* and *Staphylococcus aureus*. *Int. J. Environ. Res. Public Health* **19** (19), 12761 (2022). 36232061 10.3390/ijerph191912761 PMC9566803

[70] Wang, L. et al. The biological activity mechanism of chlorogenic acid and its applications in food industry: A review. *Front. Nutr.* **9**, 943911 (2022). 35845802 10.3389/fnut.2022.943911 PMC9278960

[71] Abonyi, D. O. et al. Biologically active phenolic acids produced by Aspergillus sp., an endophyte of Moringa oleifera. *Eur. J. Biol. Res.* **8** (3), 157–167 (2018).

[72] Bahram, M. & Netherway, T. Fungi as mediators linking organisms and ecosystems. *FEMS Microbiol. Rev.* **46** (2). (2022). 10.1093/femsre/fuab058 PMC8892540 34919672

[73] Sułkowska-Ziaja, K. et al. Natural compounds of fungal origin with antimicrobial activity-potential cosmetics applications. **16** (9). (2023). 10.3390/ph16091200 PMC10535449 37765008

[74] Hashem, A. H. et al. Bioactive compounds and biomedical applications of endophytic fungi: a recent review. *Microb. Cell. Fact.* **22** (1), 107 (2023). 37280587 10.1186/s12934-023-02118-x PMC10243280

[75] Zhu, T. et al. New Rubrolides from the marine-derived fungus Aspergillus terreus OUCMDZ-1925. *J. Antibiot.* **67** (4), 315–318 (2014). 10.1038/ja.2013.135 24326339

[76] Singh, A., Kumar, M. & Salar, R. K. Isolation of a novel antimicrobial compounds producing fungus Aspergillus Niger MTCC 12676 and evaluation of its antimicrobial activity against selected pathogenic microorganisms. *J. Pure Appl. Microbiol.* **11** (3), 1457–1464 (2017).

[77] Chowdappa, S. et al. Detection and characterization of antibacterial siderophores secreted by endophytic fungi from Cymbidium aloifolium. *Biomolecules* **10** (10), 1412 (2020). 33036284 10.3390/biom10101412 PMC7600725

[78] Witasari, L. D. et al. Antimicrobial activities of fungus comb extracts isolated from Indomalayan termite (Macrotermes Gilvus Hagen) mound. *AMB Express*. **12** (1), 14 (2022). 35142937 10.1186/s13568-022-01359-0 PMC8831673

[79] Guo, C. et al. Discovery of a dimeric zinc complex and five cyclopentenone derivatives from the sponge-associated fungus Aspergillus ochraceopetaliformis. *ACS Omega*. **6** (13), 8942–8949 (2021). 33842764 10.1021/acsomega.0c06218 PMC8028006

[80] Liu, Y. et al. A new antibacterial Chromone from a marine sponge-associated fungus Aspergillus sp. LS57. *Fitoterapia* **154**, 105004 (2021). 34339802 10.1016/j.fitote.2021.105004

[81] Li, H., Fu, Y. & Song, F. Marine aspergillus: a treasure trove of antimicrobial compounds. *Mar. Drugs*. **21** (5), 277 (2023). 37233471 10.3390/md21050277 PMC10222851

[82] Sharma, S. & Mohler, J. Microbial biofilm: A review on formation, infection, antibiotic resistance, control measures, and innovative treatment. **11** (6). (2023). 10.3390/microorganisms11061614 PMC10305407 37375116

[83] Shrestha, L., Fan, H. M., Tao, H. R., & Huang, J. D. (2022). Recent strategies to combat biofilms using antimicrobial agents and therapeutic approaches. Pathogens, 11(3), 292.‏ 10.3390/pathogens11030292 PMC8955104 35335616

[84] Yassein, A. S., Hassan, M. M. & Elamary, R. B. Prevalence of lipase producer Aspergillus Niger in nuts and anti-biofilm efficacy of its crude lipase against some human pathogenic bacteria. *Sci. Rep.* **11** (1), 7981 (2021). 33846447 10.1038/s41598-021-87079-0 PMC8041791

[85] Hamed, A. A. et al. Isolation and antimicrobial assessment of crude extract from *Aspergillus* sp. SO12 isolated from a marine source. *J. Basic. Environ. Sci.* **11**, 1–9 (2024).

[86] Machado, F. P. et al. Prenylated phenylbutyrolactones from cultures of a marine sponge-associated fungus Aspergillus flavipes KUFA1152. *Phytochemistry* **185**, 112709 (2021). 33636575 10.1016/j.phytochem.2021.112709

[87] Lobo, V. et al. Free radicals, antioxidants and functional foods: impact on human health. *Pharmacogn Rev.* **4** (8), 118–126 (2010). 22228951 10.4103/0973-7847.70902 PMC3249911

[88] Chandimali, N. et al. Free radicals and their impact on health and antioxidant defenses: a review. *Cell. Death Discovery*. **11** (1), 19 (2025). 39856066 10.1038/s41420-024-02278-8 PMC11760946

[89] Yang, L. J. et al. Antimicrobial and antioxidant polyketides from a deep-sea-derived fungus Aspergillus versicolor SH0105. *Mar. Drugs*. **18** (12), 636 (2020). 33322355 10.3390/md18120636 PMC7764742

[90] El-Neekety, A. et al. Molecular identification of newly isolated non-toxigenic fungal strains having antiaflatoxigenic, antimicrobial and antioxidant activities. *Der Pharm. Chem.* **8**, 121–134 (2016).

[91] Wang, C. et al. Purification, structural characterization and antioxidant property of an extracellular polysaccharide from Aspergillus terreus. *Process Biochem.* **48** (9), 1395–1401 (2013).

[92] Dacrory, S. Anti-proliferative, antimicrobial, DFT calculations, and molecular Docking 3D scaffold based on sodium alginate, chitosan, neomycin sulfate and hydroxyapatite. *Int. J. Biol. Macromol.* **270** (Pt 1), 132297 (2024). 38744365 10.1016/j.ijbiomac.2024.132297

[93] Dacrory, S. et al. Chitosan/cellulose nanocrystals/graphene oxide scaffolds as a potential pH-responsive wound dressing: tuning physico-chemical, pro-regenerative and antimicrobial properties. *Int. J. Biol. Macromol.* **278**, 134643 (2024). 39128733 10.1016/j.ijbiomac.2024.134643

[94] Mohamed-Ezzat, R. A., Hashem, A. H. & Dacrory, S. Synthetic strategy towards novel composite based on substituted pyrido [2, 1-b][1, 3, 4] oxadiazine-dialdehyde chitosan conjugate with antimicrobial and anticancer activities. *BMC Chem.* **17** (1), 88 (2023). 10.1186/s13065-023-01005-1 PMC10373407 37496066

